# The evaluation of the short-term and long-term hydroxychloroquine therapy on ECG parameters

**DOI:** 10.1097/MD.0000000000039039

**Published:** 2024-08-09

**Authors:** Seyit Uyar, Mehmet Kök, Ayşe Ayan, Muhammet Ali Coşkuner, Gökhan Köker, Nizameddin Koca

**Affiliations:** a Department of Internal Medicine, University of Health Sciences, Antalya Training & Research Hospital, Turkey; b Department of Internal Medicine, University of Health Sciences, Bursa Şehir Training & Research Hospital, Turkey.

**Keywords:** arrhythmias, COVID-19, hydroxychloroquine, QT prolongation

## Abstract

Amidst the COVID-19 pandemic, hydroxychloroquine (HCQ) was widely administered despite limited data on its safety and efficacy. This study assesses the acute and chronic impacts of HCQ on electrocardiography (ECG) parameters alongside the effects of azithromycin and levofloxacin coadministration in patients with COVID-19. A comprehensive analysis was conducted on 109 COVID-19 patients receiving HCQ, with or without Azithromycin and/or Levofloxacin, and 51 long-term HCQ-treated Sjogren’s syndrome (SS) patients. ECG parameters, including QTc interval, were meticulously evaluated against a control group of 109 COVID-19 patients without HCQ treatment. HCQ monotherapy, in combination with Levofloxacin, significantly prolonged the QTc interval in COVID-19 patients compared to controls. Notably, the combination of HCQ and Azithromycin demonstrated a mitigated impact on QTc prolongation. Long-term HCQ use in SS patients did not significantly affect QTc intervals, illustrating a distinct safety profile from short-term use in COVID-19 treatment. HCQ’s impact on QTc prolongation is influenced by therapeutic context, coadministered drugs, and patient demographics. The findings underscore the necessity of cautious HCQ use, particularly in acute settings like COVID-19, where monitoring and consideration of drug interactions and patient-specific factors are critical.

## 1. Introduction

HCQ, an antimalarial medication, has been employed for an extended duration in the therapeutic management of a range of rheumatic conditions, including rheumatoid arthritis (RA), systemic lupus erythematosus (SLE), and primary Sjogren’s syndrome (SS), owing to its immunomodulatory properties. It is employed in these medical conditions based on evidence indicating its ability to delay or prevent organ damage and its observed antithrombotic properties.^[1]^

HCQ is typically a well-tolerated medication, and gastrointestinal side effects frequently occur during short-term usage. Furthermore, there are other primary adverse effects, including glucose-related irregularities, dermatological reactions, neuropsychiatric incidents, retinopathy, hemolysis, and cardiotoxicity. Although HCQ has been linked to the development of cardiomyopathy and valve-related disorders in long-term use, as well as hypotension in the context of short-term administration, cardiac conduction irregularities can manifest in both conditions.^[2]^ In vivo studies and animal models demonstrated that HCQ inhibits the inward rectifying potassium, sodium, and L-type calcium channels. These actions that stabilize the cell membrane can also widen the QRS interval, slow down or block atrioventricular nodal conduction, and prolong the QT interval.^[3]^

FDA revoked HCQ’s emergency use authorization for COVID-19, citing unlikely benefits and worries about significant adverse effects, including cardiovascular system.^[4]^ The primary objective of this research was to examine the acute and chronic impact of HCQ on the electrocardiography (ECG) parameters in COVID-19 patients and patients with SS who have been long-term users of HCQ. We also evaluated the effect of concurrent administration of Azithromycin and Levofloxacin in conjunction with HCQ on ECG parameters among patients with COVID-19.

## 2. Materials and methods

### 2.1. Study design and participants

This observational study was conducted on 109 COVID-19 patients admitted to the pandemic clinics of our hospital and initiated HCQ treatment between March and July 2020. All participants were administered an initial dose of 2 × 400 mg of HCQ, followed by a maintenance dose of 2 × 200 mg daily for 4 days, adhering to the treatment guidelines provided by the country’s Ministry of Health.^[5]^

### 2.2. Group definitions and treatment regimens

Participants were divided into 5 groups based on their HCQ-based treatment:

*Control group*: 109 COVID-19 patients pre-HCQ data.
*HCQ-Only group: 58 patients received only HCQ*.
*HCQ-azithromycin group*: 14 patients received a combination of HCQ and azithromycin.
*HCQ–levofloxacin group*: 32 patients received a combination of HCQ and levofloxacin.

An additional 5 patients who received a triple therapy of HCQ, levofloxacin, and azithromycin were excluded from the study due to the small sample size of this subgroup.

*Long-term HCQ group:* A Long-term group was established from 51 long-term HCQ patients from the rheumatology outpatient clinic for comparative purposes. These patients, diagnosed with SS, had been on a stable regimen of 2 × 100 mg HCQ daily for a minimum of 6 months and exhibited no additional systemic involvement or concurrent cardiac conditions. None of these patients were diagnosed with COVID-19 or used antiarrhythmic medications.

### 2.3. Exclusion criteria

Patients were excluded from the study based on the following criteria:

A confirmed COVID-19 diagnosis via a polymerase chain reaction (PCR) test from nasopharyngeal swabs is absent.
Lack of available ECG records before and after HCQ treatment.
Presence of known arrhythmias or abnormal sinus rhythms on admission ECG.
Use of any antiarrhythmic drugs.
History of known heart diseases.
Diagnosis of chronic kidney disease.
Abnormal serum electrolyte levels in laboratory tests.

### 2.4. ECG analysis

The electrocardiograms of all study participants were analyzed before and 1 week after the administration of HCQ. Key parameters measured included the QTc interval, PR interval, and QRS duration. The Corrected QT interval (QTc) was calculated using the Fredericia formula. All ECGs were obtained using a Nihon-Kohden ECG-9132K 12-lead ECG device.

### 2.5. Ethical considerations

The research was approved by the local ethics committee of our hospital under the code number 2020-141.

### 2.6. Statistical analysis

The data was analyzed using the IBM SPSS Software package of version 26.0 (IBM Corp, Armonk). The Shapiro–Wilk’s normality test assessed the normality of variables. Categorical variables were given as numbers and percentages, and numerical variables as mean ± standard deviation or median (minimum–maximum). Continuous variables were compared in intergroup comparisons using the parametric 2-independent samples *t* test or the nonparametric Mann–Whitney *U* test. The chi-square and Fisher-Freeman-Halton tests were used to test the differences in the proportion of the categorical variables. *P* values <.05 were considered to be significant.

## 3. Results

### 3.1. Short-term HCQ treatment effects

This section compares demographic data and electrocardiographic (ECG) findings between the short-term HCQ treatment groups and the control group, which included COVID-19 patients before any HCQ treatment (Table 1).

**Table 1:** The comparison of demographic data and ECG findings between patients using HCQ alone, HCQ + Azithromycin and HCQ + Levofloxacin to the control group.

	Control (n = 109)		HCQ (n = 58)		HCQ + Azithromycin (n = 14)		HCQ + Levofloxacin (n = 32)		*P*	*P*1	*P*2	*P*3	*P*4	*P*5	*P*6
	Median (Min–Max)	Mean ± SD	Median (Min–Max)	Mean ± SD	Median (Min–Max)	Mean ± SD	Median (Min–Max)	Mean ± SD							
Female, n (%)	49(44.9)		31(53.4)		3(21.4)		12(37.5)		.136						
Age (yr)	51(21–65)	49.46 ± 12.5	46(21–65)	45.4 ± 13.38	57(43–64)	54.64 ± 6.95	58(32–65)	55.28 ± 9.53	**.003**	.058	.280	**.018**	**.029**	**.001**	.436
Heart rate (beats/min)	79(50–117)	79.89 ± 14.72	74(50–110)	75.59 ± 13.01	77(51–94)	73.79 ± 15.45	78(52–114)	79.84 ± 13.74	.212	.063*	.149*	.987*	.656*	.149*	.192*
QTc (ms)	399(320–460)	398.4 ± 25.18	413.5(352–558)	416.26 ± 30.27	393.5(340–451)	393.71 ± 37.33	409.5(383–466)	414.41 ± 19.54	**<.001**	**<.001**	.673	**.001**	.099	.987	.120
PR (ms)	152(100–218)	151.86 ± 23.15	160(116–328)	163.88 ± 36.93	162.5(110–210)	159.71 ± 28.03	153(110–202)	151 ± 22.71	.117	**.029**	.226	.990	.892	.114	.304
QRS (ms)	88(74–146)	92.53 ± 15.39	91(74–142)	95.12 ± 16.46	102(60–146)	104.36 ± 22.65	92(78–140)	93.69 ± 11.28	.052	.233	**.020**	.118	.069	.707	.058

HCQ = hydroxychloroquine, SD = Standard Deviation.

* Independent samples *t* test, Mann–Whitney *U* test used for the other continuous variables.

*P*: Probability among the groups; *P*1: Control vs HCQ group; *P*2: Control vs HCQ + Azithromycin group; *P*3: Control vs HCQ + Levofloxacin group; *P*4: HCQ vs HCQ + Azithromycin group; *P*5: HCQ vs HCQ + Levofloxacin group; *P*6: HCQ + Azithromycin vs HCQ + Levofloxacin group.

The gender distribution across the groups was not significantly different (*P* = .136). Age differences were statistically significant among the groups (*P* = .003). Specifically, the HCQ-Levofloxacin group (mean age 55.28 ± 9.53 years) and the HCQ-Azithromycin group (mean age 54.64 ± 6.95 years) were older compared to the HCQ-Only group (mean age 45.4 ± 13.38 years) and the Control group (mean age 49.46 ± 12.5 years).

Median heart rates across treatment groups showed slight variability but did not reach statistical significance (*P* = .212). Notably, the HCQ group had a median heart rate of 74 beats/min (range 50–110), compared to 79 beats/min (range 50–117) in the Control group.

#### 3.1.1. QTc interval

Significant prolongation was observed in the HCQ-Only group (median 413.5 ms, range 352–558 ms, *P* < .001) and the HCQ-Levofloxacin group (median 409.5 ms, range 383–466 ms, *P* = .001) compared to the Control group (median 399 ms, range 320–460 ms).

#### 3.1.2. PR interval

Differences in the PR interval among the groups were not statistically significant (*P* = .117). However, the HCQ-Only group showed a prolonged PR interval (median 160 ms, range 116–328 ms) compared to the Control (median 152 ms, range 100–218 ms).

#### 3.1.3. QRS duration

Significant variation was noted in the QRS duration, particularly between the HCQ-Azithromycin group (median 102 ms, range 60–146 ms) and the Control group (median 88 ms, range 74–146 ms, *P* = .020).

### 3.2. Long-term HCQ treatment effects

This section explores the demographic and electrocardiographic (ECG) data in relation to the duration of hydroxychloroquine (HCQ) treatment, comparing long-term HCQ recipients from a rheumatology outpatient clinic to COVID-19 patients who received short-term HCQ or no HCQ treatment (Control group).

There was a significant difference in gender distribution across the groups, with the Long-term HCQ group showing a notably higher proportion of females compared to males (47 females vs 4 males), which was statistically significant (*P* < .001). The median age across the groups was comparable, showing no significant overall differences (*P* = .094). However, individual comparisons revealed slight, nonsignificant age variations between the groups (Table 2).

**Table 2:** The comparison of demographic and ECG data according to HCQ treatment duration.

	Control (n = 109)		Short-term HCQ (n = 58)		Long-term HCQ (n = 51)		*P*	*P*1	*P*2	*P*3
	Median (Min–Max)	Mean ± SD	Median (Min–Max)	Mean ± SD	Median (Min–Max)	Mean ± SD				
Female, n (%)	49(44.9)		31(53.4)		47(92.1)		<.001			
Age (yr)	51(21–65)	49.46 ± 12.5	46(21–65)	45.4 ± 13.38	51(26–71)	50.57 ± 11.43	.094	.747	**.058**	**.053**
Heart rate (beats/min)	79(50–117)	79.89 ± 14.72	74(50–110)	75.59 ± 13.01	79(54–121)	78.65 ± 13.81	.192	.613*	.063*	.236*
QTc (ms)	399(320–460)	398.4 ± 25.18	413.5(352–558)	416.26 ± 30.27	398(377–494)	402.53 ± 19.29	**<.001**	.655	**<.001**	**.001**
PR (ms)	152(100–218)	151.86 ± 23.15	160(116–328)	163.88 ± 36.93	144(112–200)	147.84 ± 19.82	**.015**	.292	**.029**	**.005**
QRS (ms)	88(74–146)	92.53 ± 15.39	91(74–142)	95.12 ± 16.46	84(70–154)	86.31 ± 13.42	**.005**	**.012**	.233	**.002**

HCQ = hydroxychloroquine, SD = Standard Deviation.

Short-term: HCQ on 5th day, long-term: HCQ > 6 mo.

* Independent samples *t* test, Mann–Whitney *U* test used for the other continuous variables.

*P*: Probability among the groups; *P*1: Long-term vs Control group; *P*2: Short-term vs Control group; *P*3: Long-term vs Short-term group.

Heart rate measurements across the groups did not demonstrate significant differences (*P* = .192). The Control group had a median heart rate of 79 beats/min (range 50–117), closely aligned with the Long-term HCQ group at 79 beats/min (range 54–121) and the Short-term HCQ group at 74 beats/min (range 50–110).

#### 3.2.1. QTc interval

The Long-term HCQ group displayed a median QTc of 398 ms (range 377–494 ms), which was not significantly different from the Control group (*P* = .655). However, this group exhibited a significantly shorter QTc interval compared to the Short-term HCQ group (median 413.5 ms, range 352–558 ms), which was statistically significant (*P* < .001 for Short-term vs Control; *P* = .001 for Long-term vs Short-term).

#### 3.2.2. PR interval

The PR interval presented differences, with the Short-term HCQ group showing a longer interval (median 160 ms, range 116–328 ms) compared to the Long-term HCQ group (median 144 ms, range 112–200 ms), reaching statistical significance (*P* = .005).

#### 3.2.3. QRS duration.

Significant variability in QRS duration was also observed; the Long-term HCQ group had a median duration of 84 ms (range 70–154 ms), significantly shorter than that in the Short-term HCQ group (median 91 ms, range 74–142 ms, *P* = .002).

## 4. Discussion

### 4.1. Cardiovascular implications of COVID-19 and HCQ treatment

COVID-19 is associated with a spectrum of cardiovascular complications, including myocarditis, acute myocardial infarction, and arrhythmias. The proposed mechanism involves the virus’s interaction with angiotensin-converting enzyme (ACE)-2 receptors, which are also expressed in cardiac tissues, potentially leading to arrhythmogenic effects.^[6]^ Our study found that HCQ, a drug known for QTc interval prolongation, increased the QT interval by 14.5 ms in patients treated with HCQ alone and by 10.5 ms with HCQ combined with levofloxacin.

### 4.2. Risk of arrhythmias and QTc prolongation

Our data align with previous findings suggesting a direct correlation between QT interval prolongation and increased Torsade de Points (TDP) risks and cardiac arrest.^[7–9]^ Although we observed 1 patient’s QTc at 558 ms, most were below 500 ms, a threshold commonly associated with elevated TDP risk.^[10]^ This suggests that while HCQ can extend QTc significantly, the risk of reaching a critical threshold is generally low in a controlled clinical setting.

### 4.3. Comparative analysis of treatment regimens

Consistent with other studies, our results demonstrate variability in QTc prolongation across different HCQ-based regimens. HCQ alone led to more pronounced QTc prolongation than combinations with azithromycin, where the QTc was notably lower.^[11–13]^ This could be influenced by the younger average age of our participants (51 years) compared to those in studies by Mancuso et al^[14]^ (70.5 years) and Rosenberg et al^[15]^ (over 60 years), suggesting a potential impact of age and baseline cardiac health on drug metabolism and the pharmacokinetics of HCQ.

### 4.4. Implications for clinical practice

Given the risk of QTc prolongation, it is essential to closely monitor this ECG parameter, particularly in hospitalized patients who are more vulnerable due to systemic illnesses and the use of multiple medications. EULAR guidelines do not recommend routine ECG monitoring for patients on antimalarial drugs for rheumatological conditions. This recommendation is based on studies showing that long-term use of HCQ does not significantly prolong the QT interval in patients with systemic lupus erythematosus (SLE) and rheumatoid arthritis (RA).^[16,17]^ Our findings align with these observations, as the ECG results for the long-term HCQ treatment group showed no significant differences from the control group.

### 4.5. Study significance and limitations

This study contributes to the growing body of evidence indicating the need for careful cardiac monitoring during HCQ treatment in COVID-19, particularly when administered at higher doses or in acute settings. The distinct cardiac responses based on treatment regimen and patient demographics underscore the complexity of managing COVID-19 patients with preexisting conditions or those receiving multiple QT-prolonging drugs.

One of the limitations of this study is the small number of patients in some treatment groups, particularly the HCQ-azithromycin and HCQ-levofloxacin groups. This smaller sample size may limit the generalizability of our findings and could potentially influence the statistical power of the study to detect minor differences between groups. Additionally, the variability in treatment regimens and baseline characteristics emphasizes the need for larger, more diverse study populations to validate and extend these findings.

## 5. Conclusion

Our findings highlight the importance of personalized medicine approaches, considering individual patient risk factors such as age, baseline QTc interval, and concurrent drug therapies to mitigate the potential adverse effects of HCQ on cardiac health. Further research with larger cohorts is necessary to confirm these results and refine guidelines for the safe use of HCQ in different clinical settings.

## Author contributions

**Data curation:** Mehmet Kök, Muhammet Ali Coşkuner, Gökhan Köker.

**Formal analysis:** Nizameddin Koca.

**Investigation:** Seyit Uyar, Mehmet Kök, Gökhan Köker.

**Methodology:** Ayşe Ayan.

**Resources:** Mehmet Kök, Ayşe Ayan.

**Software:** Muhammet Ali Coşkuner, Gökhan Köker.

**Supervision:** Seyit Uyar.

**Validation:** Nizameddin Koca.

**Visualization:** Ayşe Ayan.

**Writing – original draft:** Nizameddin Koca.

**Writing – review & editing:** Seyit Uyar.
